# Prevention Focus Relates to Performance on a Loss-Framed Inhibitory Control Task

**DOI:** 10.3389/fpsyg.2019.00726

**Published:** 2019-04-05

**Authors:** Benjamin T. Files, Kimberly A. Pollard, Ashley H. Oiknine, Antony D. Passaro, Peter Khooshabeh

**Affiliations:** ^1^Human Research and Engineering Directorate, United States Army Research Laboratory, Los Angeles, CA, United States; ^2^DCS Corporation, Los Angeles, CA, United States; ^3^Department of Psychological and Brain Sciences, University of California, Santa Barbara, Santa Barbara, CA, United States

**Keywords:** regulatory focus, training, go/no-go, feedback, gamification, inhibitory control, personalization, individual differences

## Abstract

Information framing can be critical to the impact of information and can affect individuals differently. One contributing factor is a person’s regulatory focus, which describes their focus on achieving gains vs. avoiding losses. We hypothesized that alignment between individual regulatory focus and the framing of performance feedback as either gain or loss would enhance performance improvements from computer-based training. We measured participants’ (*N* = 93) trait-level regulatory focus; they then trained in a go/no-go inhibitory control task with feedback framed as gains, losses, or control feedback conditions. Some changes in performance with training (correct rejection rate and response time) were consistent with regulatory fit, but only in the loss-framed condition. This suggests that regulatory fit is more complex than cursory categorization of trait regulatory focus and feedback framing might indicate. Regulatory fit, feedback framing, and task affordances should be considered when designing feedback or including game-like feedback elements to aid computer-based training.

## Introduction

Identical feedback can have different impacts, depending on how it is framed (Tversky and Kahneman, 1981). This is important when designing human-computer interfaces, particularly training programs, since differences in feedback framing can alter the impact of that feedback and the effectiveness of the training (Kluger and DeNisi, 1998: Hattie and Timperley, 2007). Moreover, the response to feedback varies with individual differences such as regulatory focus (Levin et al., 2002; Cesario et al., 2004, 2008). In the present study, we investigate the relationship between feedback framing and regulatory focus in the context of inhibitory control training.

Regulatory focus is a psychological construct (Higgins, 1998, 2000) that posits two main motivational foci: prevention and promotion. Prevention focus involves the motivation to avoid loss, with emphasis on obligations and responsibilities and a preference for vigilant/avoidant strategies. Promotion focus involves the motivation to achieve gains, with emphasis on aspirations and ideals and a preference for eager/approach strategies. Individuals are thought to have a fairly static trait-level regulatory focus, perhaps as a result of their relationships with caregivers growing up (Higgins and Silberman, 1998).

There are multiple methods for determining a person’s regulatory focus. In this study, we used the Higgins et al. (2001) Regulatory Focus Questionnaire (RFQ). Although it includes prevention and promotion subscales, the RFQ can also be used to determine an individual’s *predominant regulatory focus*. Individuals scoring higher on the promotion subscale than the prevention subscale are considered predominantly promotion focused; the rest are considered predominantly prevention focused. An individual’s predominant regulatory focus is used to determine whether they are in regulatory fit or regulatory mismatch for a given activity.

Regulatory fit theory states that alignment between the means of pursuing a goal (e.g., task framing) and a person’s regulatory focus increases the perceived value of an activity (Higgins, 2000). A reward for a correct response might feel more valuable for promotion-focused individuals, while avoiding loss of reward might feel more valuable for prevention-focused individuals (Higgins, 1998, 2000). Regulatory fit may increase motivation and thereby improve task performance (Higgins, 2000); however, motivation is a nebulous concept that may be insufficiently specific to capture the mechanism by which regulatory fit improves task performance (Cooper et al., 2015). Regulatory fit can also lead to changes in how a person approaches a task: Regulatory fit has been observed to lead to more exploratory and more flexible behavior (Worthy et al., 2007; Grimm et al., 2009; Maddox and Markman, 2010). Performance improvements from regulatory fit have been shown in tasks such as anagrams (Roney et al., 1995; Crowe and Higgins, 1997), math tests (Grimm et al., 2009), pseudoword recognition (Förster et al., 2003), perceptual identification (Glass et al., 2011), and others.

Much of the regulatory fit literature induces a temporary situational regulatory focus in experiment participants and considers fit based on that induced, temporary focus. One method to induce a particular temporary regulatory focus is to describe performance-based compensation prior to the experiment as gaining a cash bonus for successful completion or losing a cash bonus for unsuccessful completion (Shah et al., 1998). Another approach is to ask participants to write about either ideals or obligations to induce temporary promotion or prevention focus, respectively, before taking part in an experiment (Freitas and Higgins, 2002). Although such inductions have been shown to affect performance on the timescale of hours, their effectiveness over repeated exposures and longer timescales have not, to our knowledge, been examined. Because we are interested in unobtrusive framing effects that act upon trait-level individual characteristics, we focused on chronic regulatory focus as the determinant of regulatory fit. Here, we applied regulatory fit theory in an inhibitory control training context.

Inhibitory control describes the ability to prevent or cancel an automatic response when that response is inappropriate or incorrect (Miyake and Friedman, 2012). Inhibitory control deficits contribute to real-world problems such as alcohol abuse (Houben et al., 2011), overeating (Houben and Jansen, 2011), and committing critical shooting errors (Biggs et al., 2015). Inhibitory control can be examined using a go/no-go task (Donders, 1869). This type of task involves *go* stimuli, which require a fast, overt response (here, a button press within 500 ms) and *no-go* stimuli, which require withholding the overt response. When the overt response becomes prepotent or automatic, withholding it entails overriding or preventing that prepotent response (Logan and Cowan, 1984; Boucher et al., 2007). Prepotency can be established by increasing the frequency of go relative to no-go stimuli. Strength of inhibitory control is assessed as response times on go trials and accuracy of withholding on no-go trials.

Go/no-go task performance has been shown to improve over the course of a single session of practice (Verbruggen and Logan, 2008; Benikos et al., 2013), and go/no-go training has been used to improve inhibitory control in other contexts (Houben et al., 2011; Houben and Jansen, 2011; Biggs et al., 2015). Here, we extend the work showing the practical applications of go/no-go training by looking at whether go/no-go training can be optimized by using regulatory fit to present appropriately framed feedback.

To implement this optimization protocol, we provided feedback to participants on their accuracy (go and no-go trials) and response speed (go trials) after each trial. This feedback was framed three different ways. In the loss condition, point losses indicated performance. In the gain condition, point gains indicated performance. In the control condition, simple icons (green check mark for correct; red X for incorrect) were used to provide feedback. Participants were randomly assigned to a feedback condition, thus creating regulatory fit or mismatch.

Regulatory fit is expected to yield stronger task motivation (Higgins, 2000; Spiegel et al., 2004). Because motivation is critical to learning (e.g., Wlodkowski and Ginsberg, 2017), we expected increased motivation from regulatory fit to improve inhibitory control over the course of a practice session. We also expected this improvement to transfer to a more real-world task (a simulated patrol threat-detection task with both trained and novel stimuli). The primary outcomes of interest were improvement in go/no-go task performance and transfer performance, i.e., indicators of the *effectiveness* of the training. Motivation was assessed using the effort subscale of the Intrinsic Motivation Inventory (IMI; Ryan, 1982). The main hypothesis was that regulatory fit would enhance motivation, thereby improving training effectiveness.

## Materials and Methods

The methods and hypotheses for this study, including inclusion/exclusion criteria, sample size, and analyses, were registered prior to viewing any collected data (Files et al., 2017). Data and code for the analyses are available online at https://osf.io/wrnzq/ (Files et al., 2018). The analysis presented here was registered as an additional analysis of interest; the main analysis will be reported elsewhere. Two minor changes were made to the preregistered analysis pipeline, because in both cases the data did not meet the assumptions justifying the original choices. These changes are pointed out in context below, and results from the original analyses are included in Appendix A.

### Participants

Ninety-three participants (66 F, 27 M) met all inclusion criteria and completed the experiment. The mean age was 19.5 years (range 18-55). *A priori* power analysis was used to determine this sample size. For our findings to have implications for application, they need to represent fairly large effects. Past work in regulatory fit has generally treated regulatory focus dichotomously; in a meta-analysis of such work, regulatory fit was found to have an average effect size measured with sample-weighted, reliability-adjusted Pearson’s *r* = 0.30, 95% CI [0.25, 0.36] on behavioral outcomes (Motyka et al., 2014). Therefore, we selected a target sample size that was large enough to find an effect of about that size in an analysis that treated regulatory focus as a dichotomous variable with power 0.80. In general, leaving a continuous measure as continuous can increase statistical power relative to dichotomizing it (Cohen, 1983; MacCallum et al., 2002). Therefore, this power estimate can be considered a lower bound, because we treat regulatory focus as a continuous variable in a linear modeling approach.

The voluntary, fully informed, written consent of participants in this research was obtained as required by Title 32, Part 219 of the CFR and Army Regulation 70-25. All human subjects testing was approved by the Institutional Review Board of the U.S. Army Research Laboratory under protocol 17-017.

Participants were recruited via digital message boards associated with the community around the University of California, Santa Barbara. Interested volunteers were directed to an online pre-screening instrument that was implemented using Qualtrics software. The pre-screening instrument included questions to confirm the volunteer met inclusion criteria (normal or corrected-to-normal visual acuity, color vision, and hearing; had not previously experienced severe head trauma; and were not very susceptible to motion sickness) and a shortened version of the general regulatory focus measure (Lockwood et al., 2002).

### Procedure

In the lab, participants gave informed consent, underwent vision screening using a Snellen visual acuity test and a 14-plate Ishihara color vision test, and completed a battery of questionnaires, including the RFQ (Higgins et al., 2001). They then completed the training task, followed by a series of questionnaires about their experience of the training task, including the IMI (Ryan, 1982). Participants then completed the transfer task and a final set of questionnaires. The total time was 1 to 1.5 h.

### Apparatus

Stimuli for the training and transfer tasks were displayed on a 24-inch LCD monitor (Dell Ultrasharp U2414H) driven by a PC running Microsoft Windows 7. The training task was programmed in MATLAB using Psychophysics Toolbox version 3.0.14 (Brainard, 1997; Kleiner et al., 2007). The transfer task was created using software written in Visual Basic. Responses were collected using a 6 μs-resolution RTBox (Li et al., 2010). In-lab questionnaires were administered using a tablet computer (Windows Surface Pro 3). The experiment took place in a room that was quiet, dimly-lit, and isolated.

### Training Task

**FIGURE 1:**
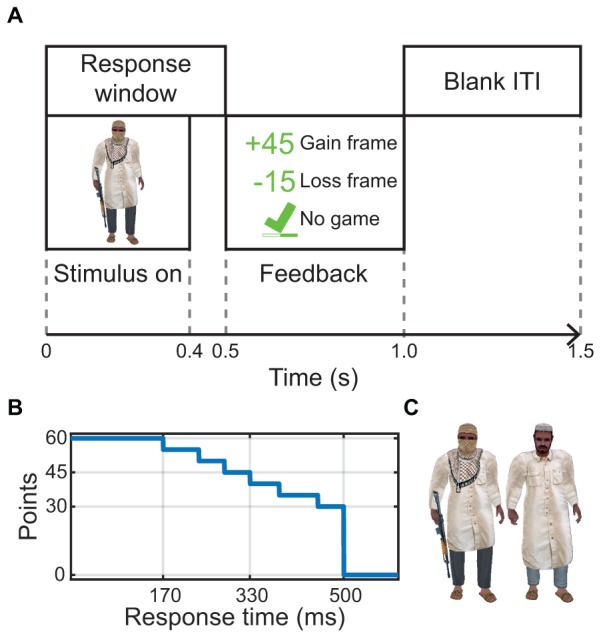
Timeline, point structure, and stimuli. **(A)** Single trial timeline of a go trial and the three kinds of feedback (gain framed, loss framed, and no points) for a correct response to a go stimulus. Each trial lasted 1.0 s and there was a 0.5 s inter-trial interval (ITI). **(B)** The curve relating response time relative to stimulus onset to the number of points achieved. **(C)** The stimuli for threat (go) and non-threat (no-go).

The training task was a go/no-go task in which participants pressed a button (or withheld button press) in response to images that appeared on a computer screen. Stimuli were computer-rendered images of two different human characters isolated from any background, with the threat (go) character designated as the one holding a rifle (Figure 1). One character was shown during each trial. The position and size of stimuli were randomized on each trial. On half of the presentations the character stimulus was mirror reversed.

Training consisted of 30 blocks of 30 trials each. Each block had 6 no-go and 24 go stimuli. The order of stimuli was determined pseudorandomly with constraints, such that there were no more than 7 go trials between no-go trials.

The timeline of a single trial appears in Figure 1A. Stimulus onset occurred at the start of the trial and was visible for 400 ms. Participants were required to respond within 500 ms of stimulus onset (i.e., less than 100 ms after stimulus offset). At 500 ms post-stimulus-onset, the feedback, which varied by condition, was displayed for 500 ms. The next trial began 500 ms after feedback offset. No variability was added to the intertrial interval. All timings were confirmed using a photodiode affixed to the bottom left corner of the display.

There were three experimental conditions that differed only in the framing of the performance feedback, which participants received after each trial and at the end of each block of the training task. Feedback was framed as point gains for successes and non-gains for failures (the *gain* condition), point losses for failures and non-losses for successes (the *loss* condition), or green check marks for successes and red Xs for failures (the *control* condition). Each participant was trained in only one of the conditions, which were assigned randomly with constraints to include approximately equal numbers of participants in each condition. Accordingly, 31 participants were assigned to the gain, 30 to the loss, and 32 to the control condition.

Visual feedback was provided at the center of the display area as part of each trial. In all conditions, the feedback indicated whether the response (or non-response) was correct or incorrect, and in the case of a response to a go stimulus, the relative response speed. In the gain and loss conditions, the feedback was delivered in the form of points. Point calculations were identical for both point-based feedback conditions. A go trial was worth up to 60 points and a no-go trial was worth 180 points. The points for the go trials depended on a piecewise-linear function of response time (Figure 1B). Responses faster than 170 ms received 60 points, with points decreasing at 5-point decrements to a minimum of 30 points for a response slower than 452 ms. Non-responses to go stimuli and responses occurring after 500 ms were awarded no points. For no-go trials, 180 points were awarded for correct non-responses, and no points were awarded for false alarms. These point values were chosen such that the value of a correct no-go non-response (180 points) was four times the average point value of a correct go response (45 points). Because go trial frequency was four times that of the no-go trials, this was intended to emphasize both trial types equally.

These point calculations favor accuracy over speed. According to the points scored relative to response time (Figure 1C), go responses would need to be 45–125 ms faster on average to make up for a single false alarm per block.

In the gain condition, points were displayed as gains (i.e., the number of points awarded), with the number colored green if the response was correct and red if the response was incorrect. In the loss condition, points were displayed as losses, calculated as the number of points awarded minus the number of points possible for that trial, with the same color coding. As examples, a response at 400 ms on a go trial would result in a green “+35” in the gain condition or a green “-25” (35–60) in the loss condition. An incorrect response on a no-go trial would result in a red “+0” in the gain condition and a red “-180” in the loss condition.

In the control condition, feedback was a green check mark for correct responses or non-responses and a red X for incorrect responses or non-responses. For go trials, the feedback also showed a graduated response time meter that was fuller as response times were faster. The resolution of this meter was matched to that of the point-based feedback such that there were 7 steps from empty to full, inclusive.

In all conditions, a vertical bar was displayed on the right side of the screen. In the gain condition, this bar began empty and filled as points were earned to indicate the cumulative gains for that block. Similarly, in the loss condition, the bar began full and emptied as points were lost to reflect the cumulative losses for that block. In the control condition, the bar began empty and incremented after each trial regardless of response to indicate progression through the block.

At the end of each block, summary feedback was displayed. Summaries included accuracy on no-go trials, accuracy on go trials, and response speed on go trials. In the gain condition, the summaries were presented as total points gained in each category; in the loss condition, the summaries were presented as total points lost in each category; and in the control condition, accuracies were displayed as percentages and the average response time (in ms) was displayed.

### Transfer Task

The transfer task was a desktop simulation of a vehicle patrol in a middle-eastern-themed town. This task was used in previous work examining performance impacts of perceived competition (Metcalfe et al., 2015). Participants were presented with the simulated forward view of a vehicle moving at fixed speed through a virtual environment with occasional obscuring fog. Five periods of fog occurred, lasting from 30 s to 2 min (mean 1 min). All participants saw an identical video presentation. The participants’ task was to watch for the appearance of each of four pre-specified stimuli and press a button with their dominant hand for stimuli that were threats and press a button with their non-dominant hand for stimuli that were non-threats.

There were 200 stimulus onsets divided among a threatening human character (the go stimulus from the training task), a non-threatening human character (the no-go stimulus from the training task), a threatening table with a tablecloth potentially concealing a roadside bomb, and a non-threatening table with no tablecloth. Stimuli were static 3D models added to the environment; as the vehicle proceeded, the participant saw each stimulus over a range of angles and sizes. Stimuli appeared for 1 s, with a 1 s response window, and the inter-stimulus interval was randomly selected from a uniform distribution of 1 to 3 s. After each stimulus offset, feedback was delivered centrally (green Y for correct, red N for incorrect, or white OO for no response).

### Analyses

Parameters of linear models were estimated, characterizing the effects of regulatory focus and feedback condition on 8 outcome measures, which were modeled separately. These included 3 measures of performance on the training task, 4 measures of performance on the transfer task, and 1 measure of subjective motivation, all described below. Promotion and prevention subscale scores from the RFQ (Higgins et al., 2001) were used as continuous predictors. The categorical variable of feedback condition was dummy-coded using two predictor variables, *g* and *l*, with *g* = 0, *l* = 0 for control. The regression model was fit with the *fitlm* command in MATLAB. The model was:

(1) $$y_{i}=\beta_{0}+\beta_{1}V_{i}+\beta_{2}M_{i}+\beta_{3}g_{i}+\beta_{4}l_{i}+\beta_{5}V_{i}g_{i}+\beta_{6}V_{i}l_{i}+\beta_{7}M_{i}g_{i}+\beta_{8}M_{i}l_{i}+𝜖,$$

where *y_i_* is the value of the outcome variable for participant *i, y_i_* is participant *i*’s prevention score, *M_i_* is participant *i*’s promotion score, and 𝜖∼𝒩 (0, σ) is error. Although the model includes a general intercept term, β_0_, no statistical tests were performed on the intercept to preserve statistical power lost due to corrections for multiple comparisons. In addition to individual coefficient tests, two pairs of regression coefficients (5 vs. 6 and 7 vs. 8) were compared with post-estimation coefficient tests (MATLAB *coefTest* method of the *CompactLinearModel* class) to test for differences in the effects of prevention and promotion in loss vs. gain framing.

All proportion measures were logit-transformed to better meet the normality assumptions in the analyses. Response times were summarized with 20% trimmed means to be robust to outliers common in response time distributions (Wainer, 1977; Wilcox, 1998). This characterization of response times differed from the pre-registered plan to analyze the parameters μ and σ of an ex-Gaussian fit to the response distribution, because the response time cutoff at 500 ms truncated the right tail of the response distribution, rendering ex-Gaussian fits unstable. Results of ex-Gaussian analysis are reported in Table A1 in Appendix A. The outcome measures were change in correct rejection (CR) rate; change in 20% trimmed mean of valid response times; IMI effort; and transfer task accuracy on the trained stimulus in fog, trained stimulus out of fog, untrained stimulus in fog, and untrained stimulus out of fog. Initial CR was also modeled to account for possible differences in initial performance on the training task, as opposed to differences in the change in training performance.

Each model entailed 11 statistical tests (8 coefficient tests, two contrasts and one omnibus). Eleven tests multiplied by 8 models yielded a total of 88 tests, controlling the False Discovery Rate (FDR; Benjamini and Hochberg, 1995; Benjamini and Yekutieli, 2001) at *q* < 0.05. Both corrected and uncorrected *p*-values are presented; 95% confidence intervals are presented without correction. For exploratory analyses that were formulated after viewing results, *p*-values are presented uncorrected.

For the training task results, initial performance was characterized by combining data from the first three blocks of training. Change in performance was characterized by computing the outcome measures over the first three blocks of training and the last three blocks of training. The initial value for each outcome measure was subtracted from the final value to obtain a difference. In pre-registration, we planned to characterize change in performance as the difference between the first three blocks and the three consecutive blocks with the best performance, but this precluded characterizing performance that got strictly worse over the course of training, and the best three blocks differed by outcome measure, complicating interpretation of effects. First-vs-best results appear in Table A2 in Appendix A.

For transfer task results, accuracy was computed as the number of correct, timely responses (RT < 1 s) divided by the number of stimulus presentations, separated by stimulus type (the characters from the training task and the untrained table stimuli) and by the presence or absence of fog. This approach treats incorrect responses, late responses (RT > 1 s), and non-responses as equally wrong.

### Data Exclusion

Prior to data collection, the following data exclusion rules were established in order to eliminate from analyses data from participants who may have misunderstood or not complied with task instructions. Data were excluded if on half or more of the blocks in the training task, the participant’s false alarm rate was greater than or equal to 83% (i.e., withheld response for no more than 1 no-go). Eight participants were excluded based on this criterion. Data were also excluded for participants with a miss rate greater than or equal to 83% on half or more training task blocks. One participant was excluded based on this criterion. For the transfer task, data were excluded if the participant never responded or only used one of the response categories. One participant was excluded due to using only the threat response button in the transfer task.

A total of 103 participants completed the experiment. The data from 10 participants were excluded based on these criteria, as described above. Data from a total of 93 participants was used for all analyses performed in this experiment.

## Results

**FIGURE 2:**
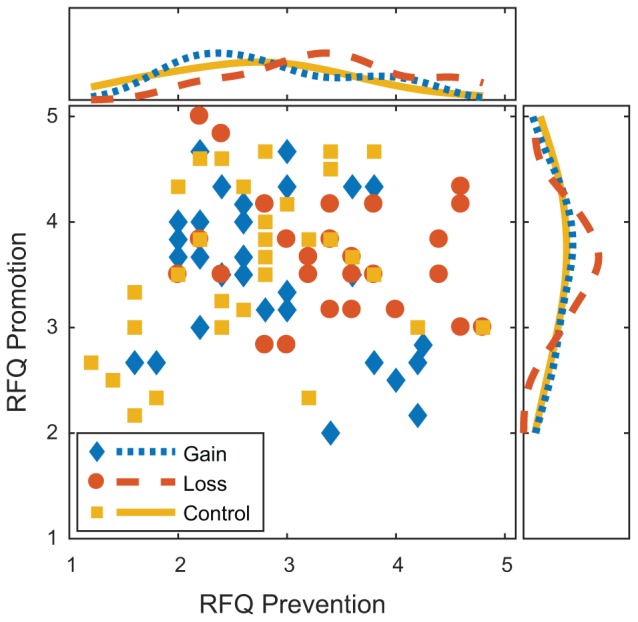
Prevention and promotion subscales for the regulatory focus questionnaire by experimental condition. Marginal plots show Gaussian smoothed histograms for prevention (top) and promotion (right) strengths to illustrate the relative frequencies of those strengths.

Prevention and promotion score distributions are shown in Figure 2. Pearson product moment correlation with bootstrapped 95% CI was *r*(91) = -0.025, [-0.245, 0.211], consistent with the claim that these are independent measures (Higgins et al., 2001; Gorman et al., 2012).

**FIGURE 3:**
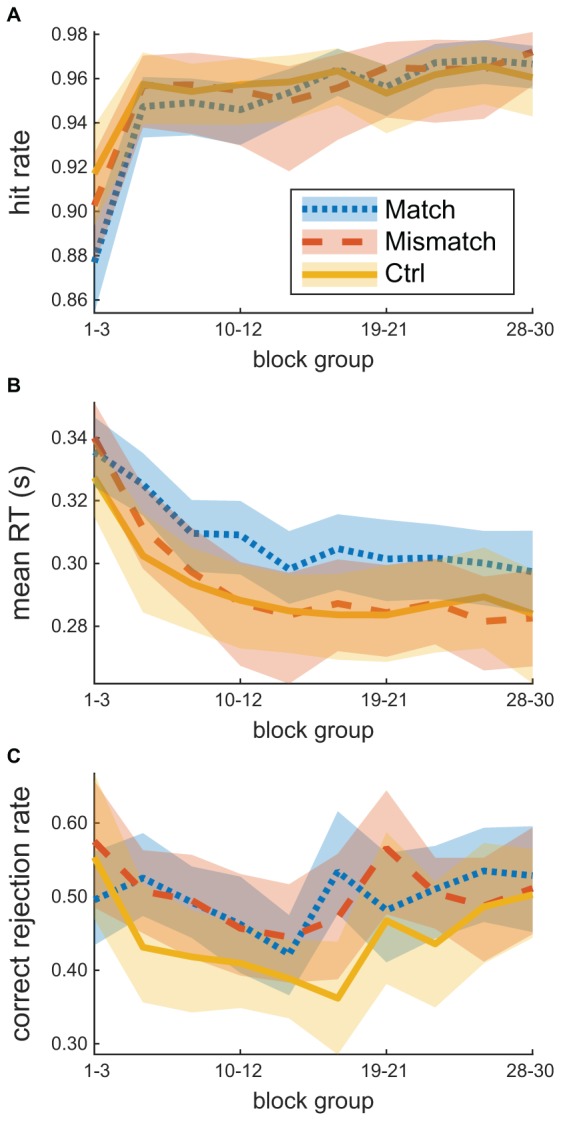
Time-courses of average training task performance. Participants were assigned to the condition that aligned (match, *n* = 37) or misaligned (mismatch, *n* = 24) with their predominant regulatory focus (Ctrl: no-points control condition, *n* = 31). Performance is summarized with **(A)** hit rate, **(B)** 20% trimmed mean response time for go stimuli, and **(C)** correct rejection rate. Summaries are computed over groups of three consecutive blocks. Shading shows a bootstrapped 95% confidence region.

Figure 3 shows how HR, 20% trimmed mean response time, and CR rate changed over the course of training, separated by match (*n* = 37), mismatch (*n* = 24), and control (*n* = 31). On average, performance improved over the course of training, but no clear advantage is apparent for participants in a condition that matches their predominant regulatory focus. Response times for participants in a feedback condition that matched their predominant regulatory focus appear slower in this sample, although the difference was not statistically significant.

**FIGURE 4:**
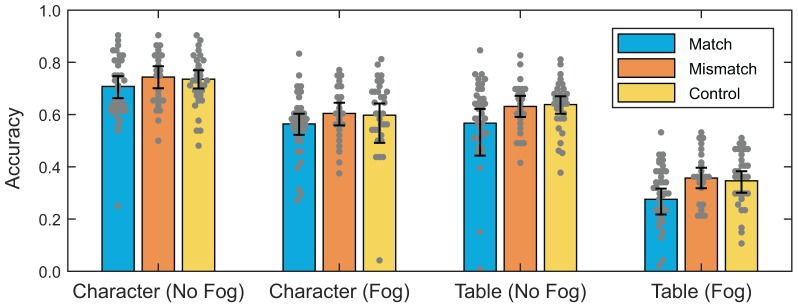
Distributions of accuracy scores for the four kinds of stimuli in the transfer task. Error bars show a bootstrapped 95% CI for the mean computed on logit-transformed accuracy. Colors indicate the training condition, with match indicating those participants who were assigned to the condition that aligned with their predominant chronic regulatory focus (*n* = 37), mismatch indicating those assigned to the condition that misaligned with their predominant chronic regulatory focus (*n* = 24), and the control condition which involved no points (*n* = 31).

Figure 4 shows performance on the transfer task, also separated by match or mismatch between condition and predominant regulatory focus and control. Again, no clear advantage is apparent due to straightforward match or mismatch.

### Linear Modeling

Linear models were fit for each of 8 outcome measures (initial CR, change in CR, change in 20% trimmed mean response time, IMI effort/importance, and transfer accuracy for trained and untrained stimuli in and out of fog) using prevention and promotion scores from the Higgins RFQ and gain/loss condition as independent variables. Coefficient estimates and corrected and uncorrected *p*-values for all analyses are provided in Tables B1, B2 in Appendix B. With FDR at 0.05, 5 of the 88 tests rejected the null hypothesis (*p* < 0.05).

**FIGURE 5:**
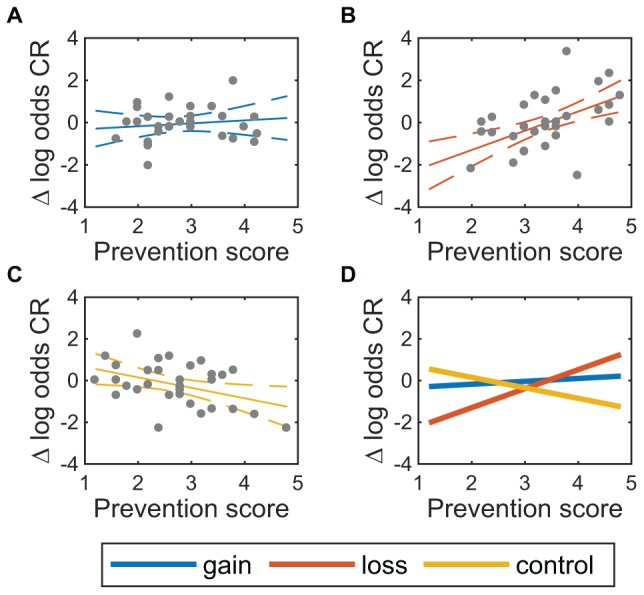
Change in correct rejection rate varies with prevention score. Change in log odds correct rejection (CR) is shown vs prevention score for **(A)** gain condition, *n* = 31, **(B)** loss condition, *n* = 30, and **(C)** control condition, *n* = 32. Solid lines show the expected mean, and dashed lines show a 95% confidence region around the expected mean. **(D)** Expected means for all three conditions.

For the change in log odds CR (Figure 5), the regression coefficient for the loss condition (β_4_ in Eq. 1) was -6.72, 95% CI [-10.58, -2.87], *t*(84) = -3.47, *p* < 0.001 (uncorrected), 0.024 (FDR correction). This shows that compared to the control condition, the loss condition reduced expected change in the logit of CR by 6.72. However, there was also a statistically significant interaction between the loss condition and the prevention score, β_6_ = 1.41, 95% CI [0.77,2.06], *t*(84) = 4.36, *p* < 0.001, 0.003 (FDR). This shows that in the loss condition compared to the control condition, the expected change in logit CR (relative to control) increases by 1.41 for each point increase in the 5-point prevention subscale. Taken together, these two results show that individuals with low prevention scores tended to worsen at withholding responses in the loss condition compared to the control condition, but individuals with high prevention scores tended to improve.

The analyses here used the control condition as baseline, which means the effects in the loss condition are not directly compared against those in the gain condition. As an additional exploratory analysis of the effect of prevention score across conditions, we carried out a coefficient contrast test to see whether the effect of prevention score was stronger in the loss condition than the gain condition. The effect of prevention score in the loss condition, β_6_ = 1.41, was marginally larger than the same effect in the gain condition, β_8_ = 0.64, *F*(1,84) = 5.06, *p* = 0.027 (uncorrected).

**FIGURE 6:**
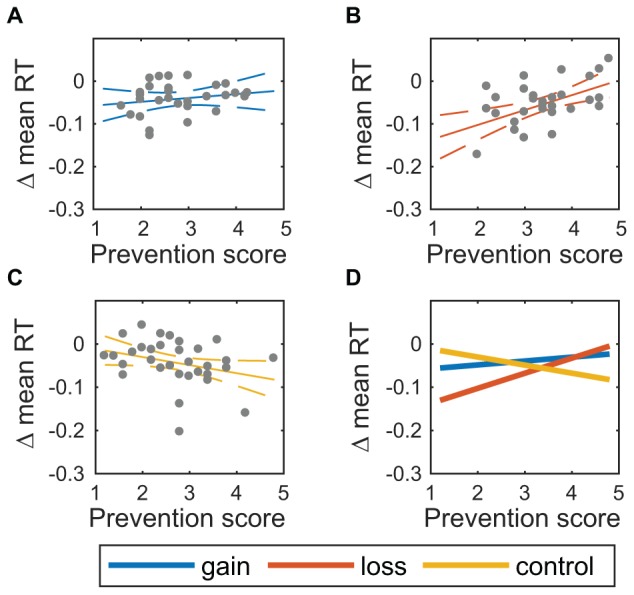
Change in response time varies with prevention score. Response time (RT), summarized as the change in 20% trimmed mean, vs. prevention score for **(A)** gain condition, *n* = 31, **(B)** loss condition, *n* = 30, and **(C)** control condition, *n* = 32. Solid lines show the expected mean, and dashed lines show a 95% confidence region around the expected mean. **(D)** Expected means for all three conditions.

For change in response time (Figure 6), the same coefficients were statistically significantly different from zero and in the same direction as change in log odds CR, showing that responses slowed in addition to becoming more accurate. The regression coefficient for the loss condition was β_4_ = -0.28, 95% CI [-0.45, -0.10], *t*(84) = -3.17, *p* = 0.002, 0.042 (FDR). The regression coefficient for the interaction of prevention score with the loss condition was β_6_ = 0.05, 95% CI [0.02, 0.08], *t*(84) = 3.67, *p* < 0.001, 0.019 (FDR). As above, an exploratory coefficient contrast test showed that the effect of prevention score in the loss condition, β_6_ = 0.05, was marginally larger than that effect in the gain condition, β_8_ = 0.03, *F*(1,84) = 2.85, *p* = 0.097.

**FIGURE 7:**
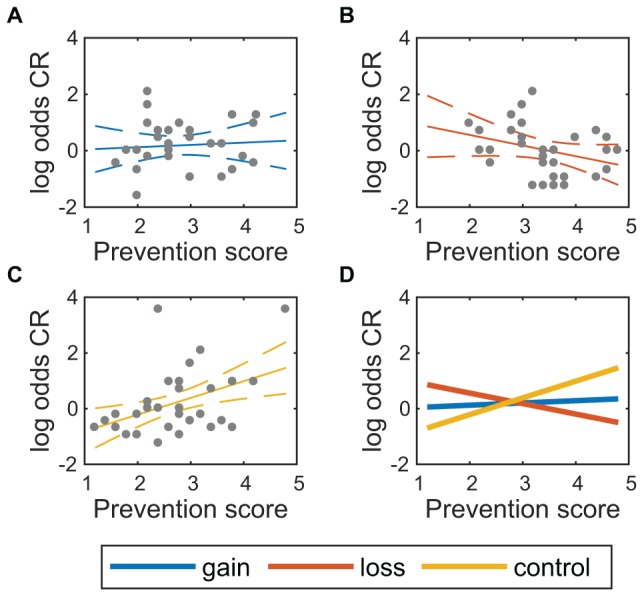
Initial correct rejection rate varies with prevention score. Initial log odds correct rejection (CR) is shown vs. prevention score for **(A)** gain condition, *n* = 31, **(B)** loss condition, *n* = 30, and **(C)** control condition, *n* = 32. Solid lines show the expected mean, and dashed lines show a 95% confidence region around the expected mean. **(D)** Expected means for all three conditions.

For initial CR rate (Figure 7), there was a statistically significant interaction of prevention score with the loss condition, β_6_ = -0.98, 95% CI [-1.60, -0.36], *t*(84) = -3.13, *p* = 0.002, 0.042 (FDR). This shows that initial CR rates were reduced in the loss condition compared to control as prevention scores increased, partially offsetting the increased gains in CR rate due to higher prevention score in the loss condition.

An exploratory analysis was run to evaluate whether the negative effect of prevention scores in the loss condition on initial CR rate offset the positive effect of prevention scores in the loss condition on change in CR rate. Although the coefficients, β_6_ = -0.98 for the initial CR rate and β_6_ = 1.41 for the change in CR rate indicate a net gain over the course of training, a more direct test is to simply run the same linear model on final CR rates. Full results of this analysis appear in Table B2 in Appendix B. The interaction of loss condition with prevention score was marginal, β_6_ = 0.44, 95% CI [-0.07,0.95], *t*(84) = 1.67, *p* = 0.098 (uncorrected). A similar exploratory test was run on final RT, and again there was a marginal interaction of loss condition with prevention score, β_6_ = 0.03, 95% CI [0.01,0.06], *t*(84) = 2.31, *p* = 0.024 (uncorrected). This analysis does not control for natural variability in the participants, so might not be very sensitive, but it does capture the overall uncertainty in final CR rate and RT.

In addition to the effects that were statistically significant after FDR correction, there were seven effects with uncorrected *p*-values < 0.05. The effects were (see Table B1 in Appendix B for details) a negative effect of prevention score on change in CR rate in the control condition, positive effects of both prevention score and loss framing on initial CR rate, and in the transfer task, positive interactions of prevention score with both gain and loss conditions on accuracy of responding to the trained stimuli both in and out of fog.

In summary, these analyses showed that in the loss condition, the prevention subscale has a negative effect on initial CR, a positive effect on change in CR, and a positive effect on change in RT. Marginal positive effects of the prevention subscale were found in accuracy for the character stimuli both in and out of fog for trainees in both the gain and loss conditions. No statistically significant effects of the promotion subscale were found.

## Discussion

The hypothesis that regulatory fit leads to increased performance resulting from training was partially supported. No effects of promotion strength on performance in the gain condition were found. However, there were reliable effects of the RFQ prevention subscale score on several of the outcome measures in the point-loss feedback condition.

In particular in the loss condition, stronger prevention focus was associated with lower initial CR rates, greater improvement in CR rates with training, and smaller improvement (and even worsening) of RTs with training. Although this represents a speed-accuracy trade-off, the points and instructions emphasized accuracy over speed, so such a trade-off was encouraged by this experiment and tended to lead to higher scores. Past work has shown that people with a stronger prevention focus (either chronic or induced) tend to weight accuracy more heavily when making speed-accuracy trade-offs (Förster et al., 2003). In the present study we only found this to be the case in the loss-framed feedback condition, suggesting that the feedback framing played an important role.

In addition to fit effects between regulatory focus and task feedback, regulatory fit theory predicts that the means available to accomplish a task can also contribute to regulatory fit. Eagerness-related strategies can lead to regulatory fit for promotion-focused individuals, while vigilance-related strategies can lead to fit for prevention-oriented individuals (Spiegel et al., 2004). In our go/no-go task that exercised inhibitory control, vigilance against responding to the no-go stimulus was a crucial element of task performance. This could explain why we saw an interaction of prevention strength and the point loss condition but not of promotion strength with the point gain condition, because both promotion focus and point gain rewards would reinforce the eagerness-based strategy of quickly responding to the go stimulus at the expense of increasing false alarm rate which was a non-optimal strategy in our task.

Although the main hypothesis of the study assumes that gains and losses have equal but opposite effects, studies of decision making have shown that in general, losses and gains are not equivalent. In a review of the decision-making literature, Yechiam and Hochman (2013b) develop a model in which losses evoke a stronger attentional response (see also Taylor, 1991). The effects of this attentional response are context-dependent and can include faster learning and less random responding. While this might predict overall better performance in the loss-framed condition (e.g., Yechiam and Hochman, 2013a), this was not observed in our study. However, because loss framing might activate individual differences more strongly than gain framing (Yechiam and Hochman, 2013b), it might be the case that loss framing amplified the effect of regulatory fit, in conjunction with increasing statistical power by reducing within-subject variability (Yechiam and Telpaz, 2013).

The increase in CR rate associated with the RFQ prevention score was partially counteracted by the relationship between RFQ prevention score and low initial CR rates. The relative sizes of the coefficients and an unplanned direct assessment showed that these partially offsetting effects still resulted in a net improvement in performance over the course of training.

Although learning was evident from performance improvements in the training task, no strong effects were obtained for the transfer task. Marginal positive effects of prevention score were obtained in the accuracy on the trained character stimuli for both loss-framed and gain-framed training (with the former marginally larger than the latter), but not control. These suggest that both point losses and point gains might interact with prevention strength to improve transfer task performance relative to no-points training, although not necessarily to the same extent.

The absence of strong transfer effects might be explained by performance on the two tasks depending on different processes. The training was meant to improve inhibitory control, but performance on the transfer task may involve considerable visual search or response selection in addition to inhibitory control. Our design precludes us from drawing any general conclusions about the effectiveness of go/no-go training for our specific transfer task. The available evidence for the benefits of inhibitory control training in a number of transfer contexts (Houben et al., 2011; Houben and Jansen, 2011; Biggs et al., 2015) suggests that enhancing go/no-go training effectiveness is likely to also improve real-world performance on some tasks.

There were also no strong effects for the IMI effort measure of motivation. This would be expected if the effects of prevention score on performance were not mediated by motivation, or if this instrument was not sensitive to a relevant change in motivational state. The lack of an effect on motivation in general is consistent with the suggestion that motivation might be an insufficiently specific account for the effects of regulatory fit (Cooper et al., 2015). Some other internal state might mediate these effects. For example, increased cognitive flexibility from regulatory fit might have resulted in improved performance in the loss condition (Worthy et al., 2007; Grimm et al., 2009; Maddox and Markman, 2010). Regardless of the mediating state, regulatory fit partially accounts for the results here.

Although we discuss a number of results in which the null hypothesis was not rejected, we make no claim that those effects are zero. With more powerful analyses or additional data, it might be possible to detect reliable, but probably relatively small, effects of promotion strength in this context, as well as transfer effects and effects on subjective motivation. A larger replication study would also be expected to reduce uncertainty of the effects estimated here.

We discuss how this result might be explained in the following sections by considering regulatory fit among regulatory focus, feedback framing, and task affordances. We also discuss practical applications of these findings to gamified training.

### Complex Regulatory Fit

We measured trait-level regulatory focus, which varies in the population, and experimentally manipulated gain and loss framing in a training task. An additional element of regulatory focus theory is that some tasks afford different kinds of successful strategies. Strategy affordances of tasks contribute to regulatory fit (Crowe and Higgins, 1997; Förster et al., 1998; Spiegel et al., 2004), such that tasks that afford an eager/approach strategy fit with promotion focus, and tasks that afford a vigilant/avoidance strategy fit with prevention focus. We did not manipulate strategy affordance in our study; all participants did the same underlying task. However, it is worthwhile to consider the strategic affordances of our tasks and our implementation of them.

In principle, success in the training task required a balance of eager and vigilant strategies. Responding quickly to go stimuli involves a positive button press response, which should be facilitated by an eager strategy. Withholding response to the no-go stimuli involves suppressing the prepotent button-press response and is facilitated by a vigilant strategy. In practice, the more frequent feedback associated with the more common go trials might have over-emphasized the eager strategy, so success depended largely on being able to implement a vigilant strategy of delaying responses to increase CR despite this emphasis. Under this reasoning, the three-way regulatory match between the participant’s prevention focus, point loss condition, and vigilant strategy afforded by the task was strong enough to overcome the reinforcement of an eager strategy.

The findings of the present study do not align with the simple regulatory fit hypothesis predicting that participants with a stronger promotion focus would perform better with gain-based feedback and participants with a stronger prevention focus would perform better with loss-based feedback. However, when the strategic affordances of the training task are considered, the results support a three-way match between prevention strength, loss-based feedback, and a task that rewards vigilant strategies. The present study provides no evidence for the opposite relationship (i.e., promotion/gain/eager), but future work could examine this possibility by applying the same design to a task that rewarded eager strategic inclinations.

Our use of chronic regulatory focus, rather than induced regulatory focus, has both advantages and disadvantages. Although strength of regulatory focus induction is not generally reported, the assumption is that these inductions produce a strong situational regulatory focus and therefore may have more power to reveal effects of regulatory fit. To our knowledge, the time-courses of induced regulatory focus effects have not been studied, nor have the effects of repeated inductions. Related work has shown that the effects of strategic orientation induction depend on a preceding induction (Woolley et al., 2013). For fit effects to be relevant to longer-term training, both topics would need to be investigated to ensure that repeated regulatory focus inductions maintain their effectiveness and have no other effects. Moreover, regulatory focus induction often takes the form of some monetary reward/penalty, and such extrinsic rewards might reduce intrinsic motivation (Deci et al., 1999). Leveraging chronic regulatory focus sidesteps those issues, enabling unobtrusive individualized feedback.

Another approach to achieve regulatory fit is to recruit participants with relatively strong chronic regulatory focus (e.g., Pincus et al., 2018) to better uncover effects of regulatory fit. We did not exclusively select participants with strong regulatory focus here, because the practical interest of the work is in improving training for all trainees. By including the full spectrum of regulatory focus strengths instead of selecting or inducing the extremes, we were able to make inferences and predictions about individuals with regulatory focus strengths across the score range.

### Implications for Gamified Learning

A practical application of this research is in the area of gamification. Gamification is the “process of making activities in non-game contexts more game-like by using game design elements” (Sailer et al., 2017, p. 372), and it is usually intended to make a non-game task more compelling in order to increase engagement with the task. The point gain and point loss framings in the present study would qualify as gamification under this definition, because point gains and losses are common game design elements.

The present work is part of a growing body of research suggesting that successful gamification should consider the individual traits of the player, since game elements that motivate some participants might demotivate others (Hanus and Fox, 2015). Previous research examining individual differences in the success of both gamified training and game contexts has shown reliable relationships between trait measures and both subjective enjoyment (Codish and Ravid, 2014; Koivisto and Hamari, 2014) and objective performance (e.g., Nagle et al., 2016).

Consistent with other work showing that gamification is not, in general, effective (Sailer et al., 2017), the present study suggests that a match in the superficial feedback elements with the trainee’s regulatory focus might not be sufficient to achieve positive outcomes from gamification. Insofar as regulatory fit theory is applicable to successful gamification, the strategic affordances of the trained task should also be taken into account when designing computer-based training. In a task with similar strategic affordances as the go/no-go training task, our findings support some specific recommendations. Based on our findings, e.g., those depicted in Figure 5, if improvement in avoiding errors of commission over the course of training is of primary concern, then trainees with very low prevention focus should be assigned to control training, trainees with high prevention focus should be assigned to loss-framed training, and trainees with intermediate prevention focus should be assigned to gain-framed training.

Without taking into account trainee traits and task structure, adding game elements to training tasks might help some trainees, but doing so could hinder others. As a practical matter, until the relationships between individual differences and the effects of gamified training can be further elucidated, gamification should be added to training with caution.

## Conclusion

In summary, we found evidence for an effect of the prevention subscale of the RFQ on performance changes in a go/no-go inhibitory control training task. These results support a three-way regulatory fit among prevention focus, loss-based feedback, and a task that rewarded vigilant strategies. This work contributes to a body of knowledge supporting future efforts to individually optimize feedback to maximize the effectiveness of training.

## Author Contributions

BF, KP, AP, and PK developed the study concept. BF and AO collected the data. BF performed the data analysis and drafted the manuscript. All authors contributed to the study design, provided critical revisions, and approved the final version of the manuscript for submission.

## Conflict of Interest Statement

AO was employed by DCS Corporation. The remaining authors declare that the research was conducted in the absence of any commercial or financial relationships that could be construed as a potential conflict of interest.
