# Supplementary material for: Prevention Focus Relates to Performance on a Loss-Framed Inhibitory Control Task
Source: Front Psychol. 2019 Apr 5;10:726. doi: 10.3389/fpsyg.2019.00726 (PMC6459883; doi:10.3389/fpsyg.2019.00726)
Supplement: Supplementary file 1 [file Table_1.DOCX]

# Appendix A

Table A1 shows statistical results of running the linear model analysis used in the main results with the pre-registered approach of fitting an ex-Gaussian distribution to the response time data using maximum likelihood estimation (Cousineau et al., 2004) and characterizing response times with the mean of the Gaussian part of the distribution, µ. This shows an increase in response time as a function of prevention strength in the loss condition, as in the main analyses. This approach was revised, because due to the response cutoff at 500 ms, response time distributions in this experiment did not have the heavy right tail typical of response times, and to which the ex-Gaussian is best suited.

Table A2 shows the full results of the pre-registered analysis approach in which changing performance is characterized as the difference between initial performance and the three consecutive blocks in which the best performance was obtained for that outcome parameter. This analysis was revised for two reasons. First, performance did not always improve and sometimes got strictly worse with training. First-vs-best comparison can never go below zero, so worsening performance could not be characterized. Second, the three blocks with the best performance were different depending on which outcome measure is considered, complicating comparisons across outcomes.

Table A1. Change in ex-Gaussian parameter µ

| source | *B* | 95% CI | | *T*(84) | *P* |
| --- | --- | --- | --- | --- | --- |
| Intercept | 0.08 |  |  |  |  |
| Prevention Strength (RFQ) | -0.02 | -0.05 | 0.01 | -1.58 | .119 |
| Promotion Strength (RFQ) | -0.02 | -0.05 | 0.02 | -0.99 | .324 |
| Loss Condition | -0.32 | -0.57 | -0.08 | -2.57 | .012 |
| Gain Condition | -0.11 | -0.32 | 0.09 | -1.08 | .283 |
| Prevention:Loss Interaction | 0.06 | 0.02 | 0.10 | 2.81 | .006 |
| Promotion:Loss Interaction | 0.03 | -0.02 | 0.09 | 1.22 | .226 |
| Prevention:Gain Interaction | 0.03 | -0.01 | 0.07 | 1.49 | .141 |
| Promotion:Gain Interaction | 0.01 | -0.04 | 0.05 | 0.29 | .775 |

Note: p-values are uncorrected for multiple comparisons.

Table A2. Analysis of change as best-minus-first

| outcome | source | *B* | 95% CI | | *T*(84) | *P* | |
| --- | --- | --- | --- | --- | --- | --- | --- |
| Change in log odds CR | Intercept | 0.66 |  |  |  |  |  |
|  | Prevention Strength (RFQ) | -0.32 | -0.59 | -0.05 | -2.36 | .021 | (.257) |
|  | Promotion Strength (RFQ) | -0.18 | -0.42 | 0.07 | -1.43 | .157 | (.501) |
|  | Loss Condition | 0.21 | -0.21 | 0.62 | 0.99 | .326 | (.657) |
|  | Gain Condition | 0.26 | -0.12 | 0.64 | 1.34 | .184 | (.536) |
|  | Prevention:Loss Interaction | 1.04 | 0.64 | 1.45 | 5.10 | .000 | (.000) |
|  | Promotion:Loss Interaction | 0.36 | -0.06 | 0.79 | 1.69 | .095 | (.429) |
|  | Prevention:Gain Interaction | 0.42 | 0.01 | 0.83 | 2.04 | .045 | (.336) |
|  | Promotion:Gain Interaction | 0.10 | -0.25 | 0.46 | 0.58 | .563 | (.780) |
| Initial log odds CR | Intercept | 0.37 |  |  |  |  |  |
|  | Prevention Strength (RFQ) | 0.51 | 0.16 | 0.86 | 2.88 | .005 | (.101) |
|  | Promotion Strength (RFQ) | 0.10 | -0.22 | 0.42 | 0.64 | .525 | (.780) |
|  | Loss Condition | -0.18 | -0.72 | 0.36 | -0.66 | .509 | (.777) |
|  | Gain Condition | -0.17 | -0.66 | 0.33 | -0.68 | .501 | (.777) |
|  | Prevention:Loss Interaction | -0.83 | -1.36 | -0.30 | -3.13 | .002 | (.079) |
|  | Promotion:Loss Interaction | -0.22 | -0.78 | 0.33 | -0.80 | .425 | (.753) |
|  | Prevention:Gain Interaction | -0.44 | -0.97 | 0.09 | -1.65 | .102 | (.440) |
|  | Promotion:Gain Interaction | -0.17 | -0.63 | 0.30 | -0.72 | .473 | (.777) |
| Change in µ | Intercept | 0.08 |  |  |  |  |  |
|  | Prevention Strength (RFQ) | 0.01 | -0.01 | 0.04 | 1.21 | .230 | (.597) |
|  | Promotion Strength (RFQ) | 0.01 | -0.01 | 0.03 | 1.09 | .279 | (.623) |
|  | Loss Condition | 0.03 | -0.01 | 0.06 | 1.54 | .128 | (.499) |
|  | Gain Condition | 0.00 | -0.04 | 0.03 | -0.27 | .786 | (.869) |
|  | Prevention:Loss Interaction | -0.04 | -0.08 | -0.01 | -2.57 | .012 | (.167) |
|  | Promotion:Loss Interaction | -0.03 | -0.06 | 0.01 | -1.45 | .152 | (.500) |
|  | Prevention:Gain Interaction | -0.02 | -0.05 | 0.02 | -0.98 | .329 | (.657) |
|  | Promotion:Gain Interaction | 0.00 | -0.03 | 0.03 | -0.27 | .790 | (.869) |
| Change in σ | Intercept | 0.03 |  |  |  |  |  |
|  | Prevention Strength (RFQ) | 0.00 | -0.01 | 0.01 | 0.51 | .613 | (.788) |
|  | Promotion Strength (RFQ) | 0.01 | 0.00 | 0.01 | 1.32 | .191 | (.539) |
|  | Loss Condition | 0.00 | -0.01 | 0.02 | 0.44 | .660 | (.820) |
|  | Gain Condition | 0.01 | -0.01 | 0.02 | 0.95 | .343 | (.657) |
|  | Prevention:Loss Interaction | 0.00 | -0.01 | 0.01 | 0.08 | .938 | (.943) |
|  | Promotion:Loss Interaction | -0.01 | -0.02 | 0.01 | -0.69 | .494 | (.777) |
|  | Prevention:Gain Interaction | 0.00 | -0.02 | 0.01 | -0.36 | .721 | (.838) |
|  | Promotion:Gain Interaction | -0.01 | -0.02 | 0.01 | -0.91 | .365 | (.682) |
| IMI effort subscale | Intercept | 4.89 |  |  |  |  |  |
|  | Prevention Strength (RFQ) | -0.20 | -0.63 | 0.22 | -0.95 | .345 | (.657) |
|  | Promotion Strength (RFQ) | 0.11 | -0.27 | 0.49 | 0.57 | .571 | (.780) |
|  | Loss Condition | 0.05 | -0.60 | 0.70 | 0.16 | .876 | (.913) |
|  | Gain Condition | 0.34 | -0.25 | 0.94 | 1.14 | .256 | (.604) |
|  | Prevention:Loss Interaction | 0.59 | -0.04 | 1.23 | 1.86 | .066 | (.383) |
|  | Promotion:Loss Interaction | 0.25 | -0.41 | 0.92 | 0.76 | .451 | (.777) |
|  | Prevention:Gain Interaction | -0.11 | -0.75 | 0.53 | -0.34 | .737 | (.838) |
|  | Promotion:Gain Interaction | -0.15 | -0.70 | 0.41 | -0.52 | .607 | (.788) |
| Log odds accuracy  Person (no fog) | Intercept | 0.99 |  |  |  |  |  |
|  | Prevention Strength (RFQ) | -0.11 | -0.33 | 0.11 | -1.00 | .319 | (.657) |
|  | Promotion Strength (RFQ) | 0.06 | -0.13 | 0.26 | 0.63 | .530 | (.780) |
|  | Loss Condition | -0.07 | -0.40 | 0.26 | -0.42 | .672 | (.821) |
|  | Gain Condition | -0.09 | -0.39 | 0.22 | -0.58 | .566 | (.780) |
|  | Prevention:Loss Interaction | 0.35 | 0.03 | 0.68 | 2.15 | .035 | (.312) |
|  | Promotion:Loss Interaction | 0.03 | -0.31 | 0.38 | 0.19 | .848 | (.893) |
|  | Prevention:Gain Interaction | 0.33 | 0.00 | 0.66 | 2.01 | .047 | (.336) |
|  | Promotion:Gain Interaction | -0.08 | -0.37 | 0.20 | -0.58 | .564 | (.780) |
| Log odds accuracy  Person (fog) | Intercept | 0.33 |  |  |  |  |  |
|  | Prevention Strength (RFQ) | -0.22 | -0.44 | 0.00 | -1.96 | .053 | (.352) |
|  | Promotion Strength (RFQ) | 0.07 | -0.13 | 0.27 | 0.72 | .473 | (.777) |
|  | Loss Condition | -0.06 | -0.40 | 0.28 | -0.35 | .730 | (.838) |
|  | Gain Condition | -0.08 | -0.39 | 0.23 | -0.54 | .591 | (.786) |
|  | Prevention:Loss Interaction | 0.47 | 0.14 | 0.80 | 2.80 | .006 | (.104) |
|  | Promotion:Loss Interaction | 0.05 | -0.29 | 0.40 | 0.31 | .759 | (.854) |
|  | Prevention:Gain Interaction | 0.37 | 0.04 | 0.71 | 2.22 | .029 | (.312) |
|  | Promotion:Gain Interaction | -0.21 | -0.51 | 0.08 | -1.47 | .146 | (.499) |
| Log odds accuracy  Table (no fog) | Intercept | 0.58 |  |  |  |  |  |
|  | Prevention Strength (RFQ) | 0.03 | -0.24 | 0.29 | 0.20 | .843 | (.893) |
|  | Promotion Strength (RFQ) | -0.05 | -0.29 | 0.19 | -0.44 | .662 | (.820) |
|  | Loss Condition | -0.16 | -0.57 | 0.24 | -0.80 | .426 | (.753) |
|  | Gain Condition | -0.33 | -0.71 | 0.04 | -1.79 | .078 | (.385) |
|  | Prevention:Loss Interaction | 0.19 | -0.21 | 0.59 | 0.96 | .341 | (.657) |
|  | Promotion:Loss Interaction | 0.12 | -0.30 | 0.54 | 0.56 | .575 | (.780) |
|  | Prevention:Gain Interaction | 0.22 | -0.18 | 0.62 | 1.09 | .281 | (.623) |
|  | Promotion:Gain Interaction | -0.11 | -0.46 | 0.24 | -0.60 | .552 | (.780) |
| Log odds accuracy  Table (fog) | Intercept | -0.66 |  |  |  |  |  |
|  | Prevention Strength (RFQ) | -0.08 | -0.31 | 0.15 | -0.69 | .489 | (.777) |
|  | Promotion Strength (RFQ) | 0.04 | -0.16 | 0.25 | 0.40 | .689 | (.822) |
|  | Loss Condition | -0.08 | -0.43 | 0.27 | -0.45 | .653 | (.820) |
|  | Gain Condition | -0.35 | -0.67 | -0.03 | -2.16 | .033 | (.312) |
|  | Prevention:Loss Interaction | 0.25 | -0.09 | 0.59 | 1.47 | .146 | (.499) |
|  | Promotion:Loss Interaction | 0.15 | -0.21 | 0.51 | 0.81 | .421 | (.753) |
|  | Prevention:Gain Interaction | 0.19 | -0.16 | 0.53 | 1.08 | .283 | (.623) |
|  | Promotion:Gain Interaction | -0.20 | -0.51 | 0.10 | -1.35 | .179 | (.536) |

# Reference

Cousineau, D., Brown, S., and Heathcote, A. (2004). Fitting distributions using maximum likelihood: Methods and packages. *Behav. Res. Methods Instrum. Comput.* 36, 742–756. doi:10.3758/BF03206555.

# Appendix B

For completeness, the full statistical results of the analyses summarized in the main document are shown in Tables B1 and B2.

Table B1. Linear model coefficients using RFQ prevention and promotion as predictors.

| outcome | source | *B* | 95% CI | | *T*(84) | *P* | |  |
| --- | --- | --- | --- | --- | --- | --- | --- | --- |
| Change in log odds CR | Intercept | 1.57 |  |  |  |  |  |  |
|  | Prevention Strength (RFQ) | -0.50 | -0.93 | -0.07 | -2.32 | .023 | (.224) | † |
|  | Promotion Strength (RFQ) | -0.11 | -0.60 | 0.37 | -0.46 | .646 | (.770) |  |
|  | Loss Condition | -6.72 | -10.58 | -2.87 | -3.47 | .001 | (.024) | * |
|  | Gain Condition | -2.63 | -5.86 | 0.60 | -1.62 | .109 | (.330) |  |
|  | Prevention:Loss Interaction | 1.41 | 0.77 | 2.06 | 4.36 | .000 | (.003) | * |
|  | Promotion:Loss Interaction | 0.68 | -0.17 | 1.53 | 1.58 | .118 | (.345) |  |
|  | Prevention:Gain Interaction | 0.64 | -0.01 | 1.29 | 1.96 | .053 | (.261) |  |
|  | Promotion:Gain Interaction | 0.28 | -0.43 | 0.99 | 0.79 | .433 | (.656) |  |
| Change in mean RT | Intercept | 0.02 |  |  |  |  |  |  |
|  | Prevention Strength (RFQ) | -0.02 | -0.04 | 0.00 | -1.92 | .059 | (.265) |  |
|  | Promotion Strength (RFQ) | 0.00 | -0.02 | 0.02 | -0.27 | .786 | (.864) |  |
|  | Loss Condition | -0.28 | -0.45 | -0.10 | -3.17 | .002 | (.042) | * |
|  | Gain Condition | -0.08 | -0.22 | 0.07 | -1.06 | .292 | (.542) |  |
|  | Prevention:Loss Interaction | 0.05 | 0.02 | 0.08 | 3.67 | .000 | (.019) | * |
|  | Promotion:Loss Interaction | 0.03 | -0.01 | 0.07 | 1.40 | .165 | (.382) |  |
|  | Prevention:Gain Interaction | 0.03 | 0.00 | 0.06 | 1.88 | .063 | (.265) |  |
|  | Promotion:Gain Interaction | 0.00 | -0.03 | 0.03 | 0.07 | .948 | (.948) |  |
| Initial log odds CR | Intercept | -1.96 |  |  |  |  |  |  |
|  | Prevention Strength (RFQ) | 0.60 | 0.19 | 1.02 | 2.88 | .005 | (.075) | † |
|  | Promotion Strength (RFQ) | 0.15 | -0.32 | 0.62 | 0.64 | .525 | (.707) |  |
|  | Loss Condition | 3.92 | 0.21 | 7.63 | 2.10 | .039 | (.238) | † |
|  | Gain Condition | 2.27 | -0.83 | 5.38 | 1.45 | .150 | (.366) |  |
|  | Prevention:Loss Interaction | -0.98 | -1.60 | -0.36 | -3.13 | .002 | (.042) | * |
|  | Promotion:Loss Interaction | -0.33 | -1.15 | 0.49 | -0.80 | .425 | (.656) |  |
|  | Prevention:Gain Interaction | -0.52 | -1.15 | 0.11 | -1.65 | .102 | (.321) |  |
|  | Promotion:Gain Interaction | -0.25 | -0.93 | 0.44 | -0.72 | .473 | (.672) |  |
| IMI effort subscale | Intercept | 5.02 |  |  |  |  |  |  |
|  | Prevention Strength (RFQ) | -0.24 | -0.74 | 0.26 | -0.95 | .345 | (.584) |  |
|  | Promotion Strength (RFQ) | 0.16 | -0.40 | 0.73 | 0.57 | .571 | (.723) |  |
|  | Loss Condition | -3.39 | -7.86 | 1.09 | -1.51 | .136 | (.366) |  |
|  | Gain Condition | 1.49 | -2.25 | 5.24 | 0.79 | .429 | (.656) |  |
|  | Prevention:Loss Interaction | 0.70 | -0.05 | 1.45 | 1.86 | .066 | (.265) |  |
|  | Promotion:Loss Interaction | 0.38 | -0.61 | 1.36 | 0.76 | .451 | (.670) |  |
|  | Prevention:Gain Interaction | -0.13 | -0.88 | 0.63 | -0.34 | .737 | (.831) |  |
|  | Promotion:Gain Interaction | -0.21 | -1.04 | 0.61 | -0.52 | .607 | (.742) |  |
| Log odds accuracy  Person (no fog) | Intercept | 1.04 |  |  |  |  |  |  |
|  | Prevention Strength (RFQ) | -0.13 | -0.39 | 0.13 | -1.00 | .319 | (.561) |  |
|  | Promotion Strength (RFQ) | 0.09 | -0.20 | 0.38 | 0.63 | .530 | (.707) |  |
|  | Loss Condition | -1.48 | -3.77 | 0.81 | -1.28 | .203 | (.425) |  |
|  | Gain Condition | -0.81 | -2.72 | 1.11 | -0.84 | .405 | (.656) |  |
|  | Prevention:Loss Interaction | 0.41 | 0.03 | 0.80 | 2.15 | .035 | (.238) | † |
|  | Promotion:Loss Interaction | 0.05 | -0.46 | 0.55 | 0.19 | .848 | (.888) |  |
|  | Prevention:Gain Interaction | 0.39 | 0.00 | 0.78 | 2.01 | .047 | (.261) | † |
|  | Promotion:Gain Interaction | -0.12 | -0.55 | 0.30 | -0.58 | .564 | (.723) |  |
| Log odds accuracy  Person (fog) | Intercept | 0.71 |  |  |  |  |  |  |
|  | Prevention Strength (RFQ) | -0.26 | -0.52 | 0.00 | -1.96 | .053 | (.261) |  |
|  | Promotion Strength (RFQ) | 0.11 | -0.19 | 0.40 | 0.72 | .473 | (.672) |  |
|  | Loss Condition | -1.98 | -4.31 | 0.35 | -1.69 | .095 | (.316) |  |
|  | Gain Condition | -0.25 | -2.20 | 1.70 | -0.25 | .803 | (.872) |  |
|  | Prevention:Loss Interaction | 0.55 | 0.16 | 0.94 | 2.80 | .006 | (.075) | † |
|  | Promotion:Loss Interaction | 0.08 | -0.44 | 0.59 | 0.31 | .759 | (.846) |  |
|  | Prevention:Gain Interaction | 0.44 | 0.05 | 0.83 | 2.22 | .029 | (.232) | † |
|  | Promotion:Gain Interaction | -0.32 | -0.75 | 0.11 | -1.47 | .146 | (.366) |  |
| Log odds accuracy  Table (no fog) | Intercept | 0.76 |  |  |  |  |  |  |
|  | Prevention Strength (RFQ) | 0.03 | -0.28 | 0.34 | 0.20 | .843 | (.888) |  |
|  | Promotion Strength (RFQ) | -0.08 | -0.43 | 0.28 | -0.44 | .662 | (.777) |  |
|  | Loss Condition | -1.47 | -4.27 | 1.34 | -1.04 | .301 | (.542) |  |
|  | Gain Condition | -0.54 | -2.89 | 1.80 | -0.46 | .648 | (.770) |  |
|  | Prevention:Loss Interaction | 0.23 | -0.24 | 0.69 | 0.96 | .341 | (.584) |  |
|  | Promotion:Loss Interaction | 0.18 | -0.44 | 0.79 | 0.56 | .575 | (.723) |  |
|  | Prevention:Gain Interaction | 0.26 | -0.21 | 0.73 | 1.09 | .281 | (.542) |  |
|  | Promotion:Gain Interaction | -0.16 | -0.67 | 0.36 | -0.60 | .552 | (.723) |  |
| Log odds accuracy  Table (fog) | Intercept | -0.60 |  |  |  |  |  |  |
|  | Prevention Strength (RFQ) | -0.09 | -0.36 | 0.18 | -0.69 | .489 | (.684) |  |
|  | Promotion Strength (RFQ) | 0.06 | -0.24 | 0.36 | 0.40 | .689 | (.798) |  |
|  | Loss Condition | -1.74 | -4.15 | 0.67 | -1.44 | .154 | (.366) |  |
|  | Gain Condition | 0.09 | -1.93 | 2.10 | 0.09 | .932 | (.942) |  |
|  | Prevention:Loss Interaction | 0.30 | -0.11 | 0.70 | 1.47 | .146 | (.366) |  |
|  | Promotion:Loss Interaction | 0.22 | -0.32 | 0.75 | 0.81 | .421 | (.656) |  |
|  | Prevention:Gain Interaction | 0.22 | -0.19 | 0.63 | 1.08 | .283 | (.542) |  |
|  | Promotion:Gain Interaction | -0.30 | -0.75 | 0.14 | -1.35 | .179 | (.394) |  |

† *p* < 0.05 (uncorrected), * *p* < 0.05 (FDR corrected)

Table B2. Omnibus and contrast test results

|  | Omnibus F test | | | Prevention contrast | | | Promotion contrast | | |
| --- | --- | --- | --- | --- | --- | --- | --- | --- | --- |
|  | *F* | *p* | *fdr* | *F* | *p* | *fdr* | *F* | *p* | *fdr* |
| change in log odds CR | 2.89 | .007 | .075 | 5.06 | .027 | .232 | 0.81 | .370 | .614 |
| change in mean RT | 2.15 | .040 | .238 | 2.82 | .097 | .316 | 1.73 | .192 | .411 |
| initial log odds CR | 1.84 | .082 | .287 | 1.92 | .169 | .382 | 0.04 | .845 | .888 |
| IMI effort subscale | 0.98 | .457 | .670 | 4.32 | .041 | .238 | 1.35 | .249 | .497 |
| Log odds accuracy Person (no fog) | 1.38 | .218 | .446 | 0.01 | .909 | .930 | 0.44 | .510 | .702 |
| Log odds accuracy Person (fog) | 1.85 | .080 | .287 | 0.28 | .596 | .738 | 2.25 | .138 | .366 |
| Log odds accuracy Table (no fog) | 1.64 | .125 | .354 | 0.02 | .899 | .930 | 1.08 | .302 | .542 |
| Log odds accuracy Table (fog) | 1.91 | .070 | .267 | 0.13 | .721 | .824 | 3.60 | .061 | .265 |
